# Carbosilane and Carbosiloxane Dendrimers

**DOI:** 10.3390/molecules14093719

**Published:** 2009-09-22

**Authors:** Chungkyun Kim, Jang Hwan Hong

**Affiliations:** 1 Department of Chemistry, Dong-A University, Busan 604-714, Korea; 2 Department of Nanopolymer Material Engineering, Pai Chai University, Daejeon 302-735, Korea

**Keywords:** dendrimer, carbosilane, siloxane, silicone, GPC

## Abstract

This review focuses on novel carbosilane dendrimers containing branches with Si-C and Si-O-C bonds. Introduction of organic moieties into the dendrimers is performed by hydrosilation of carbon-carbon double/triple bonds. Versatile organic or organometallic moieties are introduced onto the peripheral regions of dendrimers by coupling and complexation reactions, which clearly demonstrates their potential for variation.

## Introduction

**Figure 1:**
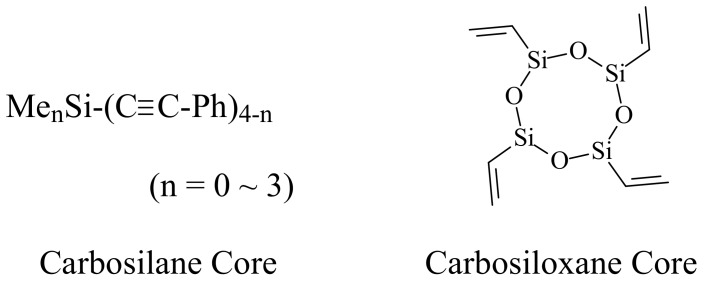
Cores for ethynylsilane (A) and siloxane tetramer cores (B).

Dendrimers of unique architecture containing a single kind of multi-functionality have been of increasing interest during the last three decades [1]. Their shapes, sizes, and structures are defined by their preparation and characterization. Dendrimer purifications are performed by traditional analytical methods such as column chromatography and recrystallization [2], but they are identified and characterized using newly developed technologies such as AFM (atomic force microscopy), STM (scanning tunneling microscopy) and PL (photoluminescence) etc. [3]. Lately, the structures of various dendrimers have been accurately confirmed as having only one class of groups on their peripheries [4]. The synthesis and characterization of dendrimers with no defects is not easy, especially in the cases of the higher generation ones. The first dendrimer, prepared by Vögtle in 1973, was a macromolecule with a distinct number of functional groups [5]. Since then many diverse dendritic molecules with versatile functional groups on their branches and/or peripheries have been prepared by many researchers [6]. The first organosilicon dendrimer was prepared by Made [7]; it has been converted into its higher generation versions by using allylic groups emanating from the central silicon atom. Diverse dendrimers having silicon-silicon (Si-Si), silicon-carbon (Si-C) and carbon-carbon triple bonds on silicon (Si-C≡C-) and siloxane bonds (Si-O), which are versatile products for state of the art material science, have been synthesized [8]. Si-C moieties have been added to the inner skeletons of organosilicon dendrimers as well as their peripheries [9]. Siloxane dendrimers with Si-O moieties were prepared before carbosilane dendrimers with Si-C moieties, although their structures were not confirmed correctly at that time [10]. The Si-O-C skeletons are introduced into siloxane dendritic macromolecules using hydrosilation and alcoholysis, as summarized in various reviews [9,11]. In this review, carbosilane dendrimers having ethynyl groups on silicon atoms (Si-C≡C-R moiety) (A) and carbosiloxane dendrimers prepared from cyclic siloxane tetramers as core moieties (Si-O-C moiety) (B) are introduced. (Figure 1).

## Carbosilane Dendrimers Based on an Ethynylsilane Core

**Scheme 1:**
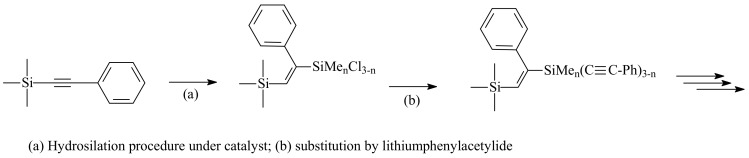
Overview of the increasing generation process of ethynylsilane dendrimers.

Ethynylsilane dendrimers with Si-C≡C-Ph skeletons on the dendritic peripheries were prepared by by diverse methods Kim *et al*. starting from a carbosilane core with one to four ethynyl groups (Scheme 1) [12]. The carbosiloxane dendrimers have carbon-carbon double bonds between two silicon atoms in their inner branches (Si-CH=CPh-Si) and/or carbon-carbon triple bonds (Si-C≡C-) on the peripheries. Carbon-carbon triple bonds are introduced onto the outer silicon atoms by the substitution reactions of lithium phenylacetylides with the chlorine atoms of chlorosilyl groups on the peripheries and carbon-carbon double bonds are introduced by the hydrosilylation of the carbon-carbon triple bonds on the outer silicon atoms. 

**Figure 2:**
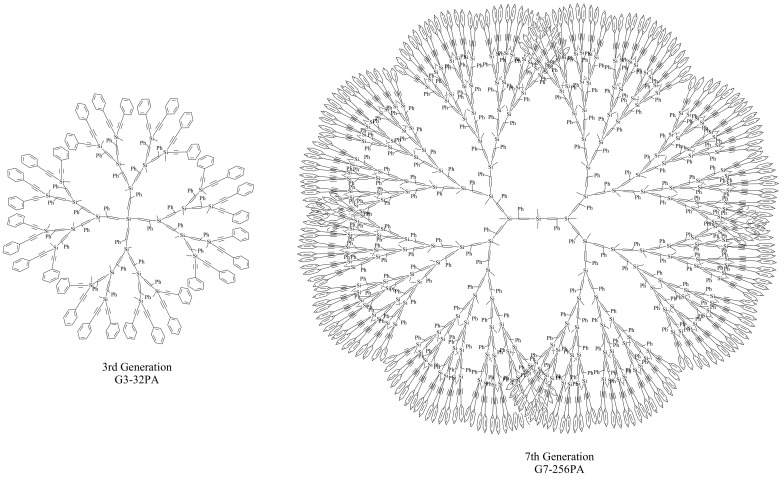
Limit generations for ethynylsilane dendrimers emanating from the (PhC≡C)_4_Si core (left) and the (PhC≡C)_2_Me_2_Si core (right).

Three different cores which have one to three phenylethynyl groups on one silicon atom at the branching point are derived from (PhC≡C)_4-n_Me_n_Si (n = 0~2) [13]. The last unimolecular generation that can be prepared is dependent on the availability of phenylethylnyl groups on each dendrimer. For four branched (PhC≡C)_4_Si as a core molecule up to the 3rd generation are prepared, with 32 emanating phenylethynyl groups, but not the 4th generation with 64 emanating phenylethylnyl groups [14]. For three branched (PhC≡C)_3_MeSi as a core molecule up to the 4th generation with 48 emanating phenylethynyl groups are prepared, but not the 5th generation with 96 phenylethynyl emanating groups [15]. For two branched (PhC≡C)_2_Me_2_Si as a core molecule up to the 7th generation with 256 emanating phenylethynyl groups has been prepared (Figure 2).

Dendrimers with bulky substituted functional groups are prepared from the alcohol moieties of 9-hydroxymethylanthracene, 2-(2-hydroxyphenyl)benzoxazole, and benzothiazole groups. The 7th generation has 256 phenylethynyl groups, 256 9-anthracenylmethoxy groups, 128 2-(2-phenoxy)benzoxazole groups, and 128 benzothiazole groups, respectively. All of carbosilane dendrimers were fully characterized by ^1^H- and ^13^C-NMR, elemental analysis, MALDI-MS, GPC, and PL (photoluminescence) [15]. Characteristically PDI (Polydispersity Index) values of the dendrimer in GPC are very narrow (1.00~1.07), which indicates that each generation of the carbosilane has a unified distribution. PL spectra of phenylethynyl- and 9-anthracenemethoxy group-substituted dendrimers show no significant change as the generation increaes from the 1st to the 7th generation. However, the PL spectra of 2-(2-phenoxy)benzoxazole group-substituted dendrimers as well as those of bezothiazole group-substituted dendrimers show a blue-shift trend as the generation increases from the 1st to the 7^th^ generation. It seems to be that the steric hindrance of the periphery affects the absorption of lights for λ_max_ according to the flexibility of the bulky functional group on the periphery. For example, the PL spectra of the 1st to the 4th generation with benzoxazole groups as well as with benzothiazole groups show a smaller λ_max_ at 347 nm and another bigger λ_max_ at 475 nm. The intensity of the smaller λ_max_ at 347 nm is increased as the generation increases, but the intensity of the bigger λ_max_ at 475 nm is decreased as the generation increases. It is reasonable that the space around Si atom becomes smaller as the generation increases. For the lower generations there is enough space around the Si atom for the bulky benzoxazole group to be in a planar geometry, in which nitrogen atom as well as oxygen atom of the benzoxazole group can be coordinated equatorially to the silicon atom. However for the higher generations the benzoxazole group is too bulky to freely move around the Si atom, therefore both the benzoxazole and phenyl groups rotate to minimize their steric hindrance [15,16] (Scheme 4).

**Scheme 2:**
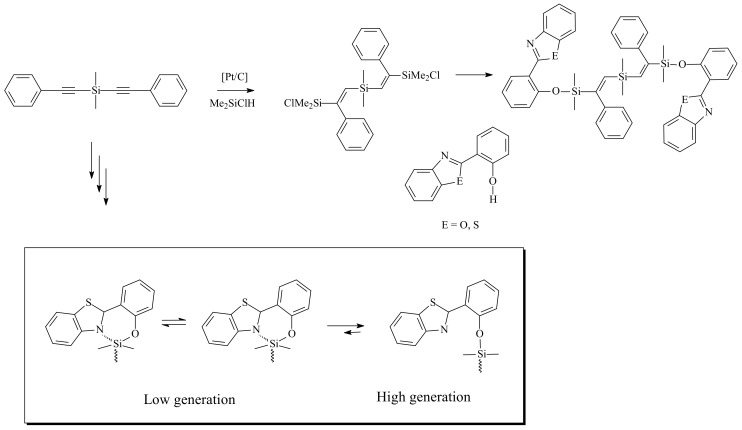
Preparation method for the 1st generation of benzoxazole and benzothiazole dendrimer and the suggested possible coordination mechanism for higher generation of the dendrimers (below).

Another novel type of carbosilane dendrimers are synthesized from 1,3,5-tris(dimethylvinylsilyl)-benzene as core (Scheme 5). The first generation with nine Cl-Si bonds is prepared by the hydrosilation of three vinyl groups on the core molecule with trichlorosilanes. Substitution reaction of the 1st generation by lithium phenylacetylide gives the 2nd generation having 27 phenylethynyl groups. However hydrosilation of the 2nd generation does not produce the 3rd generation with 81 emanating Cl-Si bonds probably due to the lack of space for them on the periphery. For the study of the less crowded dendrimer, instead of trichlorosilane, dichlorosilane is reacted with the 1st generation having nine phenylethynyl groups to give the 2nd generation with 18 Cl-Si bonds. By the two iterative reactions the 5th generation with 144 phenylethynyl groups is prepared [16].

The ethynylsilane substituted dendrimers have the feature of be easily isolated from the reaction mixtures due to the solid nature of the products. An isolation of a dendrimer with a single structure is a very important and critical point for the synthesis of its next generation with no defects. For the synthesis of the next generation it is necessary to isolate the mother dendrimer with no defects and having the correct number of branches. Even though MALDI TOF mass spectroscopy is frequently used, it has limitations for higher generation dendrimers with high molecular weight and with low stability towards the MS source. Alternatively gel permeation chromatography (GPC) is widely used for the determination of dendrimer purity [15,16,17]. For the preparation of a dendrimer with single structures only the precise synthetic reactions affording quantitative yield should be employed. For the preparation of those ethynylsilane dendrimers the simple repetitive cycles of hydrosilation and alkynylation are used. Confirmation of the complete conversion in each reaction is critical for the perfection of the structural assembly [18].

**Scheme 3:**
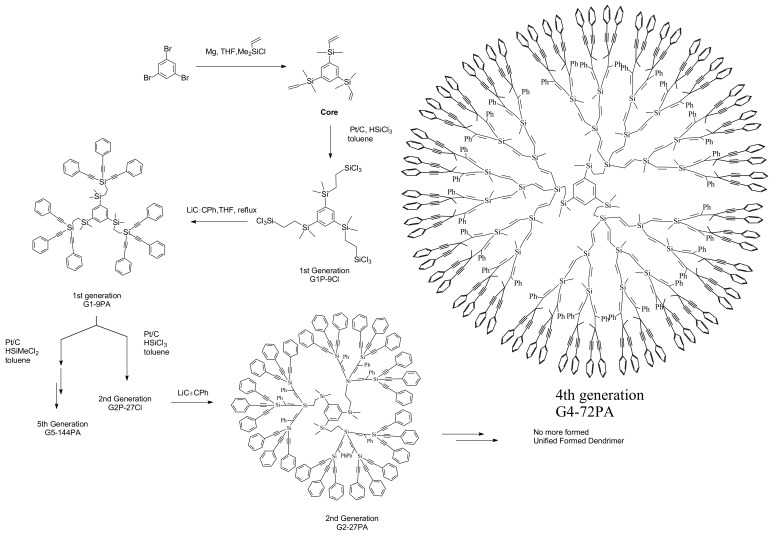
Synthetic procedure for ethynylsilane dendrimers based on a 1,3,5- tris(dimethylvinysilyl)benzene core molecule.

The ethylnyl groups of each generation is reacted with Co_2_(CO)_8_, which is reported by Kim *et al*. to give hexacarbonyl tetrahedron structures having up to 32 ethylnyl groups on the periphery [19]. The 2nd generation of ethylnyl silane and its hexacarbonyl tetrahedron structures were confirmed by MALDI TOF mass spectroscopy (Scheme 6).

Carbosilane dendrimers with silafluorenyl groups on the periphery are prepared by the reaction of 2,2'-dilithiobiphenyl with dichlorosilyl groups of carbosilane dendrimers at low temperature. The dendrimers from the 1st to the 4th generation with silafluorenyl groups are obtained with high yields. The homogenous structure of the dendrimer is measured by gel permeation chromatography (GPC) and the polydispersity index (PDI) value is measured as low at regular time intervals. The silafluorenyl moiety on the dendritic periphery is introduced with potassium fluoride ions which are stabilized by criptand [222], and its photoluminescent properties were observed [19] (Scheme 7).

MALDI TOF mass spectroscopy provides valuable information about the unimoleculirity of lower generation dendrimers; but it gives no conclusive information about its purity, so GPC was chosen to study the purity of the dendrimers. The GPC chromatogram shows very narrow peaks corresponding to that of the respective generation of the dendrimer [20].

**Scheme 4:**
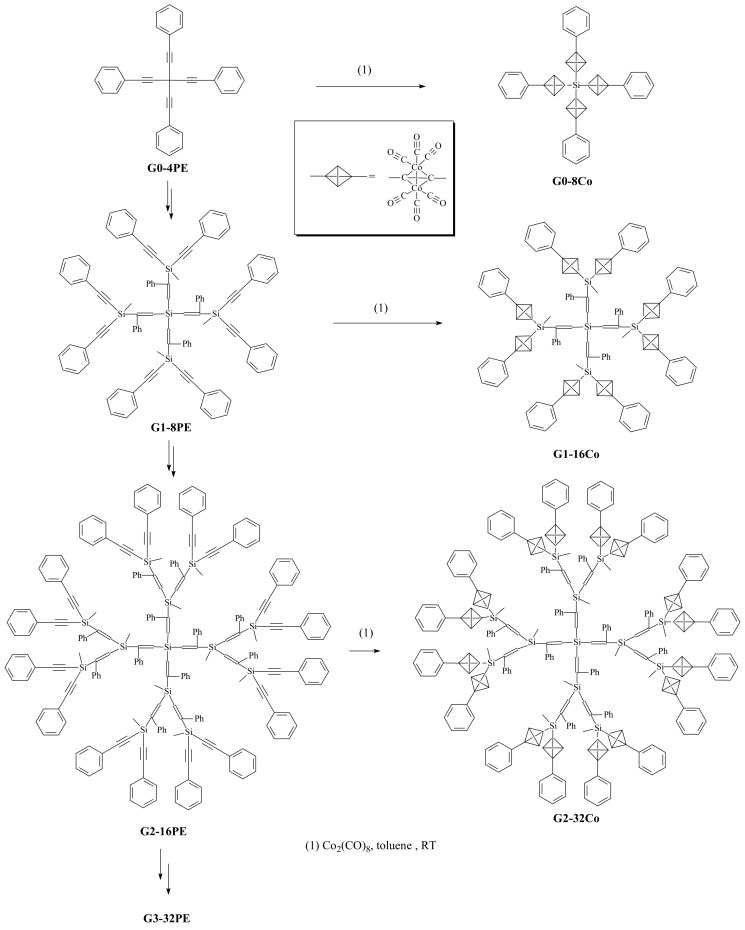
Schematic view of tetrahedrane dendrimers zeroth to 2^nd^ generation.

**Scheme 5:**
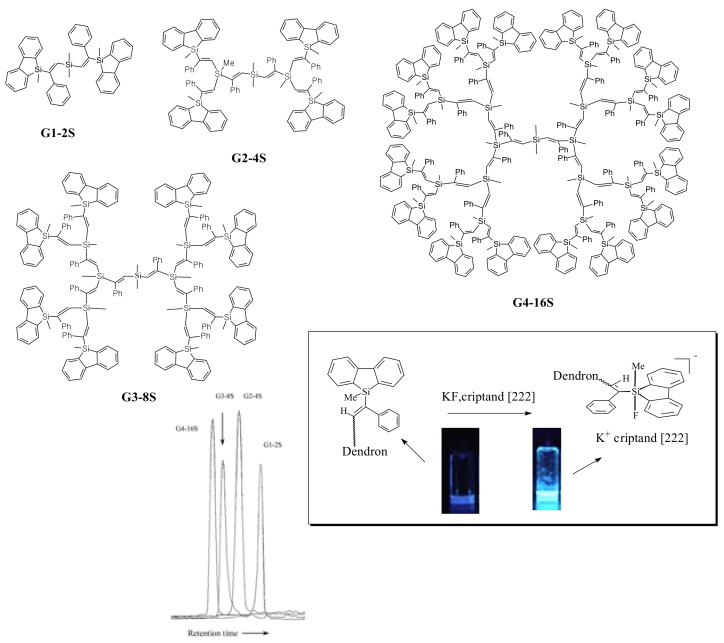
Planar view of 1st to 4th generation silafluorene dendrimers, their GPC chromatogram and photographs of their photoluminescent properties.

## Carbosiloxane Dendrimers Based on a Cyclic Siloxane Core

Dendrimers of various siloxane core moieties with characteristic branching points and peripheral groups have been prepared [9,21]. In this review the carbosiloxane dendrimers of a cyclic siloxane core (-Si-O)_4_ with Si-O-C branches are discussed [22]. The synthesis of this type of dendrimer is carried out by the divergent growing method from a (1,3,5,7-tetramethyl-1,3,5,7-tetravinyl-2,4,6,8-tetraoxacyclooctane) siloxane tetramer. The allyl group is introduced by the addition of allylmagnesium bromide after hydrosilation with chlorohydrosilanes (Me_n_SiHCl_3-n_; n = 1-3) [23] (Scheme 8).

Bulky functional groups have been introduced into the periphery of dendrimers [24]. The chlorine atom of the Si-Cl bonds on the dendritic periphery is substituted by ROH (4-hydroxypropylpyridine, 4-pyridinealdoxime, 9-hydroxymethylanthracene, 2-hydroxymethylanthraquinone, 3-hydroxycholesterol, 8-hydroxyquinoline, 4-hydroxyazobenzene, 5-(2-hydroxyethyl)-4-methylthiazole, hydroxylmethyl-crown ether) to form the respective Si-O-R bonds. The corresponding reactions are well known from many articles [25,26,27]. The Si-O-C bonds on the periphery of the above mentioned dendrimers are quite stable in wet solvents under ambient conditions [28]. A similar stability of dendrimers containing allyloxy groups is observed [29]. However the dendrimers containing aromatic alkyloxy groups, i.e. phenylmethoxy or pyridylmethoxy groups, on their peripheral silicon atoms are slightly unstable to moisture. For example, the 4th generation dendrimer with 48 1-pyridylmethoxy groups was about 20% decomposed to 1-pyridylcarbinol and unknown polymer in wet chloroform. However for the dendrimers with the long chain groups such as allyloxy, butadienyl and farnesyl groups (3, 4, 6 carbon atoms in the chain, respectively) they are quite stable after 10 days under the same conditions [30]. The stabilities of other carbosilane dendrimers with peripheral functional groups have been reported [31]. The ferrocenyl groups [32], hydroxyl groups on the peripheral layer of siloxane dendrimer [33] were prepared. A terpyridine ruthenium complex on the dendritic periphery was reported [34]. Especially for the ferrocenyl groups on dendritic periphery gas sensing abilities are demonstrated; the device was fabricated by spin coating of the dendrimer. The sensor thus produced responded linearly to various concentrations of CO gas.

**Scheme 6:**
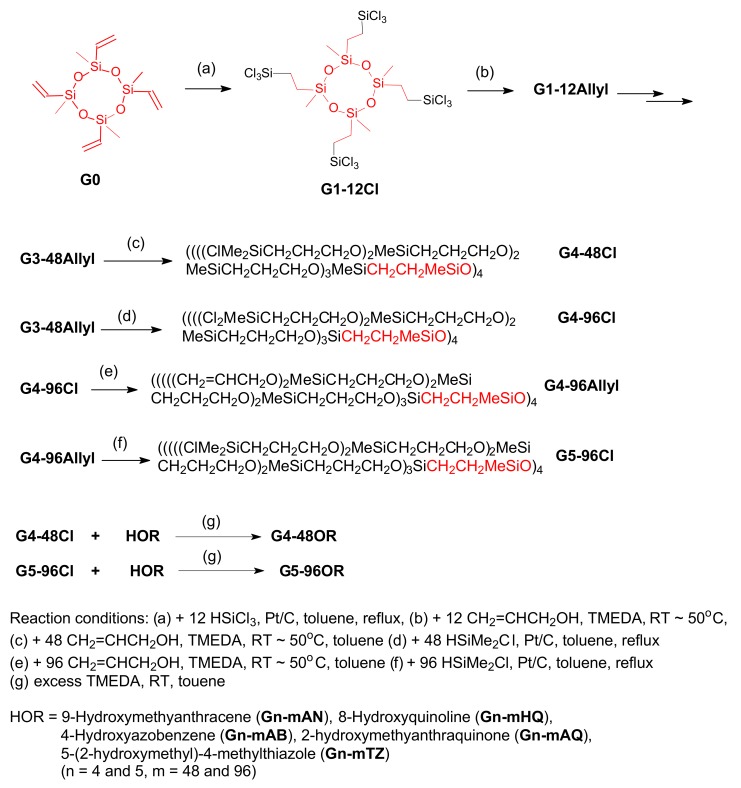
General preparation method of siloxane dendrimers.

Dendrimers with anthracene groups on the dendritic periphery have been prepared [35]. Diels-Alder reactions of anthracene on the dendritic periphery with maleimide derivatives were demonstrated. Analysis of the reaction products provided structural information on the dendrimers with no defects. The structural unimolecularity of the dendrimers is obtained from the hyperfine structure of the ^1^H- NMR spectrum and from the very low GPC polydispersity values (PDI) at the regular retention time. Furthermore, Diels-Alder reactions of the anthracenes on the dendritic periphery with 1,4-benzo-quinone and 1,4-naphtoquinone were performed with good results [36]. A dendrimer with conjugated 2,4-hexadienyl-1-oxy branches on the periphery hexadienyl was prepared [37]. Diels-Alder reactions of the conjugated dienyl groups on the dendrimer with *N*-ethylmaleimide, 1,4-naphtoquinone, and tetracyanoethene has been performed under refluxing toluene conditions. Diels Alder reaction of hexadienyl dendrimer gives the first generation with 12 bicyclo groups on the periphery but the 2nd generation is not synthesized with a single type of substituent due to the lack of enough surface to accomodate 36 enes (Scheme 9).

**Scheme 7:**
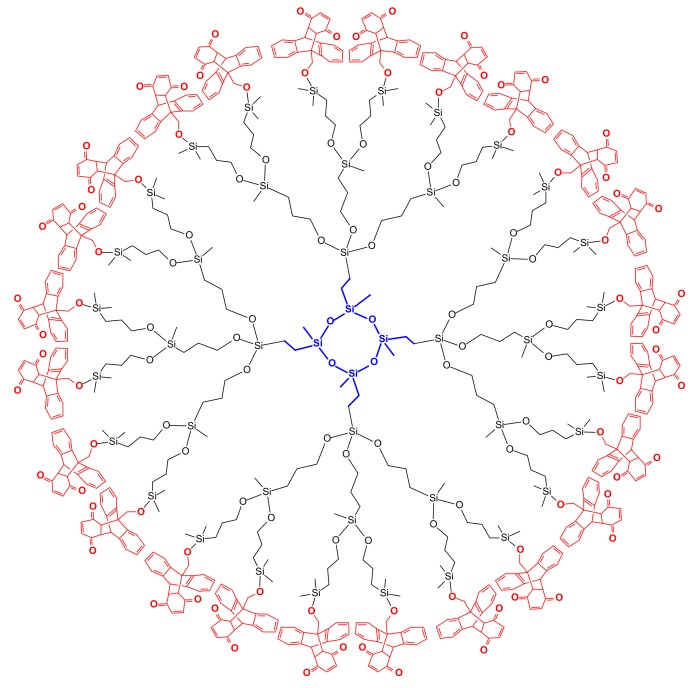
Planar view of DA products with benzoquinone (3rd generations with 24 BQ) on the dendritic periphery.

Versatile dendrimers of a siloxane core [38] and bicyclo-groups on the periphery having 96 end-groups were prepared. The structural information of the Diels-Alder product on the dendritic periphery is obtained from the hyperfine structure of the products’ NMR spectra. The purity of the product is determined by size exclusion chromatography [39]. 

A photo switching device was prepared from dendrimers with azobenzene terminal groups. The UV absorbance of the dendrimer decreases with increased irradiation time from the UV absorbance spectrum and heat treatment, however LB monolayers do not show absorbance shifts, indicating that the azobenzene dendrimer cannot be photoisomerized. Terpyridine end-functionalized dendrimers are prepared and the terpyridine groups on dendrimers are coordinated to the ruthenium ions under the mild condition to produce a paramagnetic ruthenium complex [40]

## Conclusions

This review focused on the divergent growth methods for carbosilane dendrimers with Si-C and Si-O-C moieties from carbosilane and cyclic siloxane cores. There are very few reactions for the syntheses of these dendrimers. The highly branched dendrimers are prepared from a small core up to the 7th generation and additions of various functional groups to them are reported. The siloxane dendrimers are prepared from the reaction of Cl-Si bonds of dendrimer with bulky alcohols. The repetitive cycles of hydrosilation and alcoholysis are performed for the synthesis of carbosilane dendrimers. Versatile dendritic macromolecules are prepared by using the terminal functional groups of the dendrimer. Several organic and/or organometallic moieties are introduced in the peripheral region of the carbosilane and carbosiloxane dendrimers by the simple coupling and/or complexation reactions.
